# Otherness measuring scale: design and validation for social sciences

**DOI:** 10.1186/s40359-023-01505-8

**Published:** 2024-01-18

**Authors:** Cirit Mateus, Daladier Jabba, Ana María Erazo, Ignacio Aguaded, Rodrigo Campis, Alexander Parody

**Affiliations:** 1 Research Group Direction, Colciencias – Universidad del Norte Scholar, Universidad Metropolitana, Barranquilla, Colombia; 2 Universidad del Norte, Barranquilla, Colombia; 3 Postgraduate Studies Department, Universidad Metropolitana, Barranquilla, Colombia; 4 Education Studies Department, Universidad de Huelva, Huelva, Spain; 5 Postgraduate Studies Department, Colciencias – Universidad del Norte Scholar, Universidad Metropolitana, Barranquilla, Colombia; 6 Research Department, Universidad Metropolitana, Barranquilla, Colombia

**Keywords:** Instrument validation, Coexistence, Otherness, Measurement scales, Otherness scale

## Abstract

This paper is the result of a scale validation process, applicable to social science research, which allows the constructs of otherness and coexistence and their relationship to be trasnformed into measurable, systematized variables. In turn, this scale is the product of a research project whose main objectives were (i) to demonstrate the relationship between coexistence as an independent variable and otherness as a dependent variable, and (ii) to create and statistically validate a scale to measure both variables, so to use it in applied research. The sample consisted of 600 participants. Three instruments were used: two semantic differentials and a 33-item questionnaire. The application was carried out virtually due to the SARS-CoV-2 virus. The method includes a mixed type of work, i.e., qualitative, and quantitative procedures. The results showed two factors, the other as strange or foreign and the other as equal. The final scale consisted of 10 items, with a Cronbach’s alpha of 0.86 and variance explaining 58% of the otherness.

## Introduction

By 2023, the world’s population will reach 8 billion. Today, one in 10 people lives in a city; in 35 years, it will be two in three (UNFPA, 2011) [1]. Thus, it seems that we are inexorably close to each other (Sartre, 1946) [2], so much so that physical proximity makes coexistence a global affair and an increasingly problematic situation.

Proximity creates and renews cultural problems. Žižek (2007) [3], inspired by Deleuze, even argues that the offensive of capitalist globalization provokes a split in the field of specific identities, leading to a tautological formula for exclusion. Also, Chaskin (2019) [4] explored the nature and dynamics of social inequality and exclusion in the globalisation context in China, India, South Korea, and the United States, finding a significant growing gap of social exclusion.

This contrasts with today’s knowledge society, characterised by mass access to information and greater interaction opportunities through the widespread communication devices availability, despite the uneven development of technology and social relations (Kant, 1994) [5]. This society should include educated and well-informed people, able to freely express ideas and to function properly as responsible human beings willing to interact with others.

Jenkins (2018) [6] stated that actual society is one of collective intelligence and convergence. As such, it is in groups that relating, communicating, consuming, and producing information have been transformed. Nevertheless, technology also brings the possibility of multiple actors´ simultaneous interaction, increasing the problem’s complexity.

Based on the above, the main objectives of this paper’s background research were (i) to demonstrate coexistence and otherness relationship, and (ii) to design an objective measuring scale for both. Once constructed, the 10-itme scale’s validity was empirically tested. Explaining this task and its results became the main objective of this paper.

## Literature review

### The other: stranger, foreigner or equal

Depending on the frame, the concept of the Other is explained and perceived in different ways, forcing the examination of a wide range of assessments from different authors and disciplines, so to reflect the diversity involved. This concept also inevitably refers to a social context, therefore it is useful to begin with an initial approach to the concept of society.

Touraine (1997) [7] suggested that a society can be based on solidarity and communication principles. This often implies an antagonism between the affirmation of pluralism and homogenising tendencies. Contemporary liberal societies regularly privilege diversity over universalist principles, e. i., when faith expression in institutions is forbidden to be uniform.

Meanwhile, other norms and practices tend to be subordinated to public opinion. Thus, in these societies difference and equality are not mutually exclusive, for without registered differences, dictatorships arise.

Regardless of the political line, there are three main types of encounters and relationship with the Other (Laín-Entralgo, 1968) [8]. The Other (I) as an object, e. g., reduced to an ID number; (II) as incomprehensible and inaccessible; and (III) as a neighbour, associated with love or hate. This distinction, close to psychoanalysis, includes the same: the object, the foreign and the alterity.

In this sense, Lacan (1971) [9] pointed out that the unknown subject conceals opacity and fear because we ignore it; the other is often perceived as threatening, making it unbearable. It is ignored that the same thing dwells in everyone else, so the other suffers and is fragile like everyone else.

Bauman (2005) [10], from a sociological view, proposed that human relations and the possibility of living together in globalising societies are crossed by mistrust. This is evident in the particularities of living with others in this century. In addition to highlighting the fragility of ties, this leads to a globalised anthropological crisis (Bauman, 2005) [10]. Thus, relations with the other would be mostly characterised by fragility and superficiality.

Krotz (2016) [11], from anthropology, understands the other as a member of society and not only in terms of individual or “natural” characteristics. He describes them as bearers of culture, heirs to a tradition, representatives of a community, nodes in a long-term communicative structure and insiders in a particular symbolic universe.

Consequently, the Other becomes both the result and the creator, part of a specific, unique, and unrepeatable historical process. Moreover, he adds an appreciation of otherness as a specific type of differentiation related to the experience of the strange or different, including the sense of the immigrant (Moffitt & Juang, 2019 [12]; Martínez & Muriedas, 2019) [13].

This context determines the emergence of subcultures, collectives, and ghettos, as well as the emergence of what Bauman (2012) [14] calls the architecture of fear, which is used to explain people’s relationships referred to public spaces. From this analysis, he conceptualises mysophobia and mixophilia as opposing concepts, referring respectively to the fear of exchange and the desire to mix with difference.

Today, the relation between otherness, difference and objectification continues to be discussed from different scientific disciplines, focusing on the same categories proposed by sociologists, anthropologists, philosophers, and psychoanalysts of the last century. However, social problems and their contexts are renewed. An example of this approach is the focus on creating policies that promote inclusion and acceptance of diversity, which only leads to the exacerbation of segregation.

According to Strieder and Herbert (2015) [15] the emphasis on promoting mechanisms of homogenisation and universal affirmations that sideline the strange, the different and the irrational only contributes to keeping people in cold and distant relationships. The logic of the techno-scientific paradigm supports this coldness, which encloses the world in technocracy and neglects the human dimension.

Giaccaglia et al. (2012) [16] define the encounter with the other as a universal and fundamental experience. They state that their encounter confronts the “human” with the “foreign” and that the common attitude or reaction is conflict and war, with the main difficulty being the impossibility of communication. On the contrary, trade, culture, art, and religion, among others, are factors that make communication and coexistence possible.

In this sense, Sanvicen-Torne, Moren & Molina-Luque (2017) [17] claim that it is a widely studied fact in sociology that a high degree of population diversity in a geographical space does not imply greater contact, interrelationship, understanding between groups or the appreciation of differences as a collective value, a claim that is widely accepted (Alcoverro, 2005 [18]; Deusdad, 2009 [19]; Esquirol, 2005 [20]; Garreta, 2004 [21]; Jorba, 2011 [22]; Villalobos et al., 2016) [23]. The situation is exacerbated when the other is perceived as a threat.

For example, Bekus (2017) [24] describes various representations of Poland in Belarusian culture (TV programmes, films, novels and theatre performances), thus presenting an ‘other’ whose threat to Belarusian identity is actualised in patriotic discourses. Another version can also be found in microaggressions in institutional spaces, such as schools or workplaces (Liu & Kramer 2019) [25].

There are also other conceptions of the other as ‘neighbour’ and ‘equal’ (Strieder & Herbert, 2015) [15] and still others as ‘stranger’ (Alian & Wood, 2019) [26]. The latter have shown that lived and formative - even simulated - experiences of friendship are empowering for the recognition of the Other. However, these experiences are often associated with homogenising pedagogical relationships that tend to subjugate and exclude the Other and the ‘different’.

It is also significant to highlight the importance of recognising the need for caution in the face of complexity and historical difficulties in understanding the Other (Strieder & Herbert, 2015 [15]; Skovgaard-Smith, Soekijad & Down, 2019) [27]. It is suggested that the lived experience of friendship enables the construction of positive visions of the world, the self, and the Other as individual and diverse human beings.

Coexistence in friendship considers scenarios of respect for otherness that involve the discovery of self and other as an exercise in this coexistence. In this sense, it seeks to eliminate the fixed polarity around the tension between ‘belonging’ and ‘otherness’, a binary system that creates a symbolic barrier (Hall, 2010) [28]. It can therefore be concluded that the sense of “belonging” is another dimension of coexistence that implies prejudices that prevent rapprochement with the other.

In line with this idea, recent studies have found that the organisation of spaces according to cultural issues plays a crucial role in abolishing hierarchies between groups of different origins, opening new possibilities for the sense of “belonging” (Markovich, 2018) [29]. Other proposals also point out that the effectiveness of cultural encounters is based on the identification of familiar social characters and desires (Bekerman, 2007 [30]; Shenhav & Yonah, 2005 [31]; Shohat & Stam, 2003 [32]; Liu & Kramer, 2019) [25].

Shenhav & Yonah (2005) [31] found that openness to shared resources, emotions, experiences, and aspirations is facilitated by a dialogical process that is effective when political or social positions are set aside. In this regard, Guttormsen (2018) [33] also states that the cultural theory of otherness and the other is an integral part of identity construction during intercultural encounters but has been largely neglected in intercultural management research.

This gives specific characteristics to coexistence today; on the one hand, it would be what it means to live with others: “Coexistence refers us to respect, recognition, solidarity and empathy, to the ability to identify with others, to understand their points of view” (Bayón 2017 p. 302) [34]. On the other hand, there are obstacles such as nationalism, identity issues, divisions of thought and minorities, among others, which contribute to creating an aporia around the definition proposed by Bayón [34].

Considering the above, this review shows that conceptions of the Other have not fundamentally changed, although new conflicts and forms of social interaction are emerging. Rapprochement depends largely on reaching a point of connection with the other and overcoming the barriers of problems of identity, belonging and, finally, the emphasis on difference, which has become a major obstacle to coexistence.

### Validation process

For validation, the usual steps and procedures have been followed. These include item reduction, factor extraction and the application of validity and reliability tests, as well as other statistical procedures (Boateng, Neilands, Frongillo, Melgar-Quiñonez, & Young, 2018 [35]; Hair, Gabriel, Silva, & Braga, 2019) [36].

#### Scale selection

Involves a careful systematic review of the available literature and, where appropriate, experts ´consultation in the measured area.

#### Preliminary adaptation test

The version defined in the previous phase is administered to subjects (between 10 and 15) by two or three different raters. Subjects and raters have similar characteristics to those of the final scale application scenario. Aspects related to item specificity and scale utility are also analysed (Streiner, 1993) [37].

Regarding to item specificity, their comprehensibility is considered, i.e., they should be written simply, easy to understand, avoiding technical or rarely used terms and without ambiguity or affective load. The frequency of response is also considered (without exceeding 95%, as it does not contribute to the variability of the instrument), as well as the scale ranges (if there are 5 possible answers and one is not chosen, the range should be reduced) (Sánchez & Echeverry 2004) [38].

#### Validity tests

Face validity. Two groups are paired, one of subjects being measured with the scale and another of experts who review it and decide if it measures what it proposes. This is not a statistical process but allows quantification for greater precision (Sánchez & Echeverry 2004) [38].

Content validity. This involves statistical methods, e. g., factor analysis (Nunally 1978) [39], which allows the factor structure and the items of the different factors to be known. It allows items´ elimination that do not contribute variability to the phenomenon’s measurement. At least five subjects are required for each item, and not less than 100 in total (Norman & Streiner, 1996 [40]; Sánchez & Echeverry 2004) [38].

#### Reliability test

Refers to the behaviour of the scale under different conditions, depending on the instrument itself, the time of administration and the clinician administering the measurement. Reliability measures the potential for error of an unstable instrument used under different conditions and is assessed in relation to the three conditions listed above (Sánchez & Echeverry 2004) [38]. Thus, the correlations between items, between items and factors, and between items and scale give the instrument internal consistency or homogeneity. They are measured using various procedures, including Cronbach’s alpha (Sánchez & Echeverry 2004) [38].

Intra-class correlation coefficient. “This is a better measure of reliability than the previous one because, in addition to between-subject variability, it includes in the analysis other sources of variability such as different observers, patient characteristics (also called within-subject variability) and error. It can be calculated using a statistical procedure called “repeated measures analysis of variance” (ANOVA) (Sánchez & Echeverry 2004, p. 314) [38].

## Methodology

The mixed-methods research began with 267 documents (mostly scientific papers), from a rigorous literature review on otherness and coexistence in specialised databases, i. e., Dialnet, Jstor, Redalyc, Sage, Scopus, Springer, and Web of Science. Documents were published within the 7 years prior to 2021 when the research began.

Classic texts up to 20 years old were also included. Nevertheless, all documents came from the main disciplines from which both concepts are approached, i. e., philosophy, anthropology, sociology, psychology, education, and communication.

The texts were first chronologically arranged using Excel spreadsheets and then organised according to their content, which facilitated the identification of key words, paragraphs, and extracts, so to identify units of analysis and to prepare the codebook.

Researchers carried out a content analysis based on the units identified, using ATLAS.ti software to ensure greater reliability and validity of the data and analysis. In this way it was possible to make reproducible inferences based on the information and its context, which is part of its validation (Krippendorff, 1990) [41].

### The sample

The universe consisted of adults associated or related to local universities. A convenience sample of 600 people aged between 18 and 68 was selected based on proximity. Participation was voluntary and informed consent was explicitly stated at the top of the forms. Once read, consent was formalised by clicking on the screen of the data collection device.

In terms of their characteristics 61.84% of the sample identified themselves as female and 38.16% as male, with an average age = 27.8 years and a mode = 20. University education was reported by 92.11% (different levels and programmes), of which 82.29% were students, 11.5% teachers and, to a lesser extent, university managers and administrators and relatives of participants without academic training.

### Validations

As mentioned above, semantic differentials were created upon categories and indicators resulting from the content analysis (one for Otherness and one for Coexistence). These were validated through analysis of variance and internal consistency. This supported the assumption of both as variables and at the same time identified the indicators.

The Kaiser, Meyer & Olkin (KMO) test determined the partial correlation coefficients between the dimensions of the Otherness and Coexistence variables, being its value between 0 and 1. This indicates a high relationship between variables, as it tends towards 1. The application of the tools yielded a result of 0.88, which is “remarkable” with a tendency towards “very good”.

The applicability of the variables´ factor analysis was also checked using Bartlett’s test of sphericity, which indicates that the test is feasible if the significance value (p) is < 0.005. The *p*-value obtained was 0.000 with a determinant of 0.019. This shows a linear relationship between the variables, so factor analysis is appropriate.

Next, results of the instruments´ application were analysed with SPSS V.25, while the KMO and Bartlett’s sphericity tests were again applied, followed by an ANOVA. Of the 400 semantic differences, 200 were used for each variable to examine the direct effects between coexistence and otherness. All these results led to the construction of the 33-item questionnaire.

Regarding the sample, Lloret-Segura (2014) [42] indicates that optimal conditions exist when saturations are higher than 0.70 and the number of variables per factor is adequate - at least 6 items per factor. Thus, a sample of 150 or 200 cases allows for accurate coefficient estimates in exploratory factor analysis (EFA) (MacCallum, Widaman, Zhang and Hong, 1999 [43]; Preacher, Wichman, MacCallum and Briggs, 2008) [44]. There is even evidence that 100 cases are sufficient when there are three factors with three or four items each, or when there are more items and factors, but the agreement is more remarkable than 0.80 (Bandalos & Finney, 2010 [45]; Costello & Osborne, 2005 [46]; Guadagnoli & Velicer, 1988) [47].

In view of the above, the validation was carried out with 200 participants, none of whom took part in both procedures. The questionnaire was validated twice. First, before use, by a panel of experts - three PhD psychologists. This validation consists of “the informed opinion of people with experience in the field who are recognised by others as experts. [They are qualified and can provide information, evidence, judgements, and evaluations” (Escobar and Cuervo (2008, p. 29) [48].

The consensus of at least two judges determines the validity of the items (Pedrosa, Suárez Álvarez, & García-Cueto, 2013) [49]; a third judge was introduced in anticipation of possible disagreements between the first two. Table 1 shows the judges’ rating categories and indicators.

**Table 1:** Evaluation criteria for experts’ validation

Factors	Assessments
**Sufficiency** Items belonging to the same category are sufficient to obtain its measurement.	The items are not sufficient to measure the dimension/category. The items measure some aspect of the dimension, but do not correspond to the total dimension. Some items should be increased to be able to assess the dimension completely. The items are not sufficient
**Clarity** The item is easily understood, i.e., its syntax and semantics are adequate.	The item is not clear. The item requires a lot of modification or a very large modification in the use of words in terms of their meaning or their order. A very specific modification of some terms is required. The item is clear, has adequate syntax and semantics.
**Coherence** The item has a logical relationship with the dimension or indicator it is measuring.	The item has no logical relationship to the dimension/category it is measuring. The item has a tangential relationship to the dimension/category it is measuring. The item has a moderate relationship to the dimension/category it is measuring. The item is completely related to the dimension/category it is measuring.
**Relevance** The item is essential or important, i.e., it should be included.	The item can be removed without affecting the measurement of the dimension/category. The item has some relevance, but another may be including what it measures. The item is relatively important. The item is very relevant and should be included.

Fuente: Escobar & Cuervo, 2008 [48]

The judges’ evaluation led to wording adjustments, some signifiers changes, and placement of some items in a different dimension of the variable. A hypothesis test (Student’s t-test) was then applied to the judges’ responses to statistically support compliance with the minimum score.

After the adjustments, the questionnaire was administered virtually due to the Covid-19 pandemic. The communalities test was then used to determine which indicators were least well explained by the model. Based on these results, the number of factors obtained and their sufficiency to adequately represent the dimension of the variables was decided. No factor was rejected, as all items obtained a value higher than 0.30 after the test. However, factor analysis reduced the data and enabled to find uniform groups of items of a given variable from a large set.

Similar groups were constructed from closely correlated items, to create exclusive dimensions between groups of items. In the first round of analysis, seven dimensions were extracted and reduced, either because their factor loadings were less than 0.40 or because they assessed a dimension for which they were not designed. Finally, two dimensions were defined: The Other as Stranger or Foreigner and The Other as Equal. The other as object was subsumed under the stranger or foreigner dimension; otherness or difference was discarded as it did not achieve factor loadings above 0.40.

As this was a new instrument (questionnaire) without precedent of this type, a principal component analysis was applied with the aim of using it in applied research. A new set of factors was regrouped due to a linear composition of the original factors. Two dimensions were identified, (Stranger/Foreigner - Equal). Their interpretation can be very complex, so the study was based on a VARIMAX rotation because of its suitability when the number of components is reduced. Thus, each rotated component showed correlations with only a few dimensions.

At this point, the Corrected Total Item Correlation was applied to determine the linear correlation between the item and the total scale score, and to identify the magnitude and direction of this relationship. This indicates that subjects will tend to score the same on the item and the scale. Six items with an item-to-total ratio of less than 0.2 were discarded and it was concluded that they did not measure the same as each other and it did not make sense to combine them into a total score.

Finally, internal consistency was determined with Cronbach’s alpha, based on the mean of the correlations between each item. This test is a great advantage because it allows the reliability of the scale to be improved by excluding items. In the process of designing and refining the scale, six items were excluded because they did not meet the condition of correlation and the result was a test with 10 items and a value of 0.869.

## Results

### Categories and indicators for coexistence and otherness as variables

**Fig. 1:**
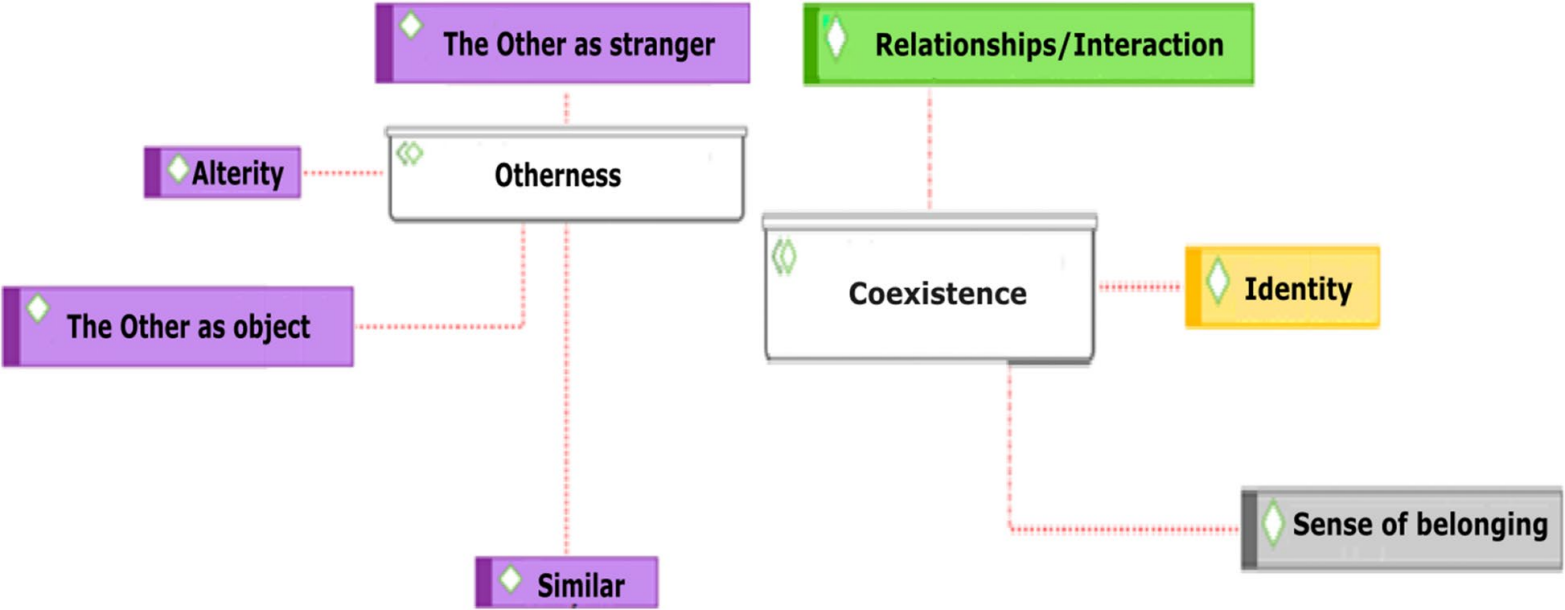
Network of relations for Otherness and Coexistence. Source: Self - made with ATLAS.ti

First, dimensions obtained from the content analysis are presented. These allowed both constructs´ operationalisation and transformation into variables. In turn, dimensions were established with literature review’s data on coexistence and otherness. Occurrences – indicated in square brackets – determined three emerging categories for the variable Coexistence (Interaction/Relationships - 89, Appearances, Identity - 57 and Sense of Belonging - 79) and four for the variable Otherness (The Other as Stranger - 89, Similar - 91, The Other as Object - 35 and Alterity - 63) as seen in Fig. 1.

The same process led to the identification of indicators for each dimension as shown in Table 2.

**Table 2:** Dimensions, indicators and references for variables Coexistence and Otherness

**Coexistence**		
**Categories (Dimensions)**	**Indicators**	**Authors**
***Sense of Belonging***	**Inclusion Minorities Regionalism**	Kymlicka, 2006 [50]; Rodríguez et al., 2006 [51]; Etxeberria, 2003 [52]; Giménez, 2003 [53]; Garreta, 2004 [21]; Lévy, 2004 [54]; Pierre, 2004 [55]; Shenhav & Yonah, 2005 [31]; Jenkins, 2018 [6]; Deusdad, 2009 [19]; Rifkin et al., 2002 [56]; Barabas, 2014 [57]; Mossiere, 2016 [58]; Sanvicen-Torne et al., 2017 [17]; Chaskin, Lee & Jaswal, 2019 [4]; Liu & Kramer, 2019 [25].
***Identity***	**Diversity** **Unit** **Ideologies**	Giménez, 2003 [53]; Córdova, 2003 [59]; Blondel, 2018 [60].
***Relationships/ Interaction***	**Equity** **Conflicts** **Respect** **Intolerance**	Ajzen & Fishbein, 1980 [61]; Giménez, 2003 [53]; Etxeberria, 2003 [52]; Pérez-Latre, 2004 [62]; Bekerman, 2007 [30]; Said, 2015 [63]; Ramos & Igartua, 2014 [64]; Barabas, 2014 [57]; Bayón, 2017 [34]; Villalobos et al., 2016 [23]; Polhuijs, 2018 [65]; Najmanovich, 2018 [66].
***Otherness***		
**Dimension / Categories**	**Indicators**	**Authors**
***Representation of the other as a stranger, foreigner, or enemy***	**Strange** **Foreign** **Alien** **Enemy**	Laín-Entralgo,1968 [8]; Lacan, 1971 [9]; Kojeve, 1982 [67]; Todorov, 1991 [68]; Miranda & Castillo, 2001 [69]; Hercman, 2016 [70]; Lévinas, 2001 [71]; Villoro, 1998 [72]; Deusdad, 2009 [19]; Garreta, 2004 [21]; Esquirol, 2005 [20]; Jorba, 2011 [22]; Gudiño-Bessone, 2011 [73]; Bauman, 2012 [14]; Giaccaglia et al., 2012 [16]; Villalobos et al., 2016 [23]; Bekus, 2017 [24]; Sanvicen-Torne, et al., 2017 [17]; Abramovitch, 2019 [74]; Gonçalves, 2019 [75]; Moffitt & Juang, 2019 [12]; Martínez & Muriedas, 2019 [13]; Civila, Romero y Aguaded, 2020 [76].
***Representation of the other as an object***	**Indifferent** **Tool** **Obstacle**	Laín-Entralgo, 1968 [8]; Lacan, 1971 [9]; Kojeve, 1982 [67]; Todorov, 1991 [68]; Miranda & Castillo, 2001 [69]; Hercman, 2016 [70].
***Representation of the other as a neighbour or equal***	**Compatriot** **Understandable** **Equal** **Close**	Laín-Entralgo, 1968 [8]; Lacan, 1971 [9]; Touraine, 1997 [7]; Bekerman, 2007 [30]; Shohat & Stam, 2003 [32]; Shenhav & Yonah, 2005 [31]; López-Miguel, 2016 [77]; Sanvicen-Torne et al., 2017 [17]; Hercman, 2016 [70]; Markovich, 2018 [29]; Liu & Kramer, 2019 [25]; Alian & Wood, 2019 [26].
***Representation of the other as alterity***	**Different** **Divergent** **Varying**	Freud, 1917 [78]; Lacan, 1971 [9]; Villoro, 1998 [72]; Garreta, 2004 [21]; Bauman, 2005 [10]; Alcoverro, 2005 [18]; Esquirol, 2005 [20]; Rodríguez et al., 2006 [51]; Gudiño-Bessone, 2011 [73]; Deusdad, 2009 [19]; Jorba, 2011 [22]; Strieder & Herbert, 2015 [15]; Krotz, 2016 [11]; López-Miguel, 2016 [77]; Guttormsen, 2018 [33]; Alian & Wood, 2019 [26]; Skovgaard-Smith, Soekijad & Down, 2019 [27].

Source: Authors

### Results of the construction of the semantic differentials obtained with the dimensions and indicators found in the content analysis

From these data, a semantic differential was elaborated for each variable (coexistence and otherness). These new data collection tools were evaluated using the criteria listed in Table 1 and the procedures and tools described in the methodology. The results of this task were as follows.

#### Qualitative results of the expert assessment

Each judge evaluated the instruments adapted to their initial indications independently, ignoring each other’s judgements and identities, with the following results:

**Sufficiency:** All agreed that the items measured the dimension they were intended to assess.

**Clarity:** The first round of scoring resulted in an adjustment of the wording (first person) and occasional omission of the subject, considering the need for self-administration.

**Coherence:** Following the judges’ suggestions, statements related to beliefs, values, affects and emotional actions or reactions were eliminated, and only cognitive aspects were addressed. This ensured greater proximity between the statements and their intended elements.

**Relevance:** Reviewers agreed that the proposed items were relevant to the questionnaire and should all be included.

Finally, and in response to the reviews, care was taken in the use of generic terms in Spanish. All this led the reviewers to conclude that the level of coherence between items, statements and variables was satisfactory.

#### Quantitative results of the expert assessment

Numerically, the validation target was a minimum compliance of 75% corresponding to an average of 3.75 in judges’ evaluation and which was exceeded in all three cases (J1–3.9 / J2–4.2 and J3–3.9). It resulted in an overall mean of 4.04, corresponding to a compliance of 80.6%. (Table 3).

**Table 3:** Summary statistics

Judges - Experts	Count	Average	Standard deviation	Coefficient of Variation (%)	Min.	Max.
J1	33	3.921	0.193	4.928	3.400	4.000
J2	33	4.246	0.655	15.423	2.000	5.000
J3	33	3.945	0.246	6.243	3.400	4.800
Total	99	4.037	0.44	10.916	2.000	5.000

Source: Authors

It is worth mentioning that the scores given to each item were similar, with a percentage of less than 20% in the coefficient of variation of each judge and in the overall coefficient of variation, indicating that the good results are consistent across all items.

#### Semantic diffentials´ statistical validation

##### Relationship between coexistence and otherness´ perception

Statistical analysis of the results of the application of semantic differentials revealed a direct relationship between coexistence and perceived otherness as follows:

The KMO is a statistical test whose results show the partial correlation coefficients between the dimensions of the variable. Table 4 (see above) shows that there is a relationship (value of 0.58). On the other hand, the Otherness Differential was 0.808, which also indicates a relationship, as shown in Table 5.

**Table 4:** KMO and Bartlett test results for Coexistence

Kaiser-Meyer-Olkin sample adequacy measurement	
0.584	
Bartlett’s sphericity test	
Approx. Chi-square	322.377
LG	45
Sig.	0.000

Source: Authors

**Table 5:** KMO and Bartlett test results for otherness

Kaiser-Meyer-Olkin sample adequacy measurement	
0.808	
Bartlett’s sphericity test	
Approx. Chi-square	773.303
LG	45
Sig.	.000

Source: Authors

Statistical tests of differentials produced values indicating that variables used are linearly related.

##### Correlation of variables

Consequently, the correlation between the variables was analysed using a simple linear regression model, thus generating an equation that demonstrates the linear relationship between the variables. In this case, the independent variable coexistence is identified as X and the dependent variable otherness is identified as Y; with this statistical procedure, a linear model is obtained that explains the relationship between the variables in the entire population covered by the data obtained in the sample.

The Table 6 shows the correlation rate of both variables (R-0.997) and a square R of (0.954), indicating a high correlation between Coexistence and Otherness, as its result is over 80%. Statistical correlation analysis is used when (i) none of the variables has been controlled, (ii) both have been measured, and (iii) it is desired to know if they are related.

**Table 6:** Simple linear regression model without intercept (Model resume^c,d^)

Model		Sum of squares	LG	Quadratic mean	F	Sig.
1	Regression^a^	381,395.019	1	381,395.019	4167.226	.000^c^
	Residual	18,212.981	199	91.523		
	Total	399,608.000^d^	200			

Source: Authors

a. Predictors: Coexistence.

c. Dependent variable: Otherness

d. Linear regression through origin

Also, the calculated significance was of 0.000c. Since *p*-value is significant, it can be accepted that there is a correlation, and its magnitude is that which indicates the coefficient. *P*-value is highly significant, and the quadratic mean shows that the model explains the relationship between the variables by 91.523%, as shown in Table 7:

**Table 7:** ANOVA

Model		Sum of squares	LG	Quadratic mean	F	Sig.
1	Regression^a^	381,395.019	1	381,395.019	4167.226	.000^c^
	Residual	18,212.981	199	91.523		
	Total	399,608.000^d^	200			

Source: Authors

a. Dependent variable: Otherness

c. Predictors: Coexistence

d. This total sum of squares is not corrected for the constant because the constant is zero for regression through the origin

##### Otherness scale statistical analysis

The instrument is a questionnaire designed from scratch for use in applied research. Its statistical validity was verified with an exploratory factor analysis (EFA), using Principal Component Analysis and Varimax rotation.

First, a set of factors was formed from the linear composition of the original, called dimensions (two), and then the internal consistency of the factors was verified. At the factor level, commonalities, factor loadings, item-factor correlations, linearity assumptions and sample adequacy were analysed based on the determinant, Bartlett’s sphericity test and KMO. Cronbach’s alpha (α) was used to verify the internal consistency of the factors (see Table ).

The Otherness measuring scale obtained a bifactor structure with eigenvalues greater than 1 (one labelled as stranger or foreigner and the other as similar). Finally, because of the EFA, a 10-item scale was obtained from an initial 33 items. Table 8 presents the values obtained for the communality, the corrected item-total correlation, the factor loadings of each item and Cronbach’s alpha [79] to determine the internal consistency of the scale.

**Table 8:** Statistical properties of the Otherness Scale

	ÍTEM	Communality	C. Item-total Corrected	The other as a stranger or a foreigner	The other is similar
**1**	If I see someone who dresses or wears their hair in strange ways, it causes me discomfort	0.68	0.75	0.76	___
**2**	It seems threatening to let immigrants enter the country	0.53	0.63	0.71	___
**3**	Strangers, generally speaking, seem annoying to me	0.67	0.74	0.79	___
**4**	I am afraid of being in a place with strangers	0.52	0.63	0.68	___
**5**	I believe that others corrupt identity and culture	0.53	0.64	0.70	___
**6**	I think strangers can be very harmful	0.52	0.63	0.67	___
**7**	I generally feel that others are indifferent to me	0.56	0.63	0.75	___
**8**	I believe that others may be able to harm their fellow human beings to achieve their achievements	0.47	0.48	0.67	___
**9**	I think that people can establish bonds of friendship, even if they belong to racial minorities or identity groups	0.60	0.42	___	0.73
**10**	I think people tend to group with other people	0.70	0.27	___	0.84

Source: Authors

As mentioned above, factor analysis reduces data to find uniform groups of items of a variable. Thus, positively correlated items create exclusion dimensions between groups of items (Lloret-Segura 2014) [42].

It should be recalled that two factors were delimited and the category The Other as Object was subsumed under Foreigner or Stranger. The category Alterity was excluded because of the factorial measures of its items (below 0.40).

This results in the scale of 10 items, each with a commonality of more than 0.30 and a factor loading of more than 0.40. Finally, the corrected item-total correlations showed values above 0.30 for the final scale.

The closeness of the value in the KMO test to 1 is directly proportional to the relationship between the variables. If KMO ≥ 0.9, the test is excellent; if KMO ≥ 0.8, the test is remarkable. Median for KMO ≥ 0.7; low for KMO ≥ 0.6; and very low for KMO < 0.5 (Lloret-Segura 2014) [42]. The KMO for this scale was 0.887, indicating good correlation and sampling adequacy.

Bartlett’s test of sphericity determines the feasibility of factor analysis and, in this case, gave an approximate chi-square of 767.467 with gl = 45 and *p* < 0.000, indicating a high degree of correlation between the items. The determinant was 0.019, which means that the variables are linearly related and factor analysis can be applied (Table 9).

**Table 9:** KMO Test and Bartlett Sphericity

Kaiser-Meyer-Olkin sample adequacy measurement	
0.887	
Bartlett’s sphericity test	
Approx. Chi-square	767.467
LG	45
Sig.	.000

Source: Authors

Overall, the *Otherness Scale’s* bifactor structure explains the variance of *otherness* by 57.91%.

As seen in Table 8, the Cronbach’s alpha coefficient (α) tests the internal consistency based on the average of item-item correlations and is advantageous for improving the reliability of the scale by applying item exclusion (Bonett & Wright 2015) [79]. Regarding the reliability of the 10-item scale, an adequate level of 0.863 and 10 items were obtained.

## Discussion

The scientific method of logical positivism and its heirs requires an observational conceptualisation that would force the foundations of social science concepts to be laid in terms of measurable variables. This would increase the possibility of replication and thus the generalisability of the results obtained.

For determining the frequency of an event related to coexistence and otherness, or identifying the factors related to these concepts, it is necessary (i) an adequate selection of theories that address the issue, (ii) the establishment of dimensions, and (iii) indicators that define the object of study. Finally, a careful scale for measuring the characteristics, i.e., the variables in question, must be defined.

Measurement with scales is used when the object of measurement cannot be precisely defined or when it is very complex, either because it has a variety of characteristics (because it has multiple theoretical approaches that lack consensus within the scientific community) or, as in the case of psychological, social, educational, and other phenomena, because it is neither directly observable nor easy to measure.

Coexistence and otherness have traditionally been treated as constructs analysed and described in terms of semantic and/or semiotic units from phenomenological or hermeneutic perspectives (Craig, 1995) [80]. This could be understood as an obstacle to the generation of scientific knowledge. Odysseos (2007) [81] for example, catalogues this treatment as negligent because it is a cutting-edge transnational problem.

It is worth recalling here the Kantian vision of the divergent pace of development between social sciences and technology, due to the compartmentalisation of knowledge in the disciplines dealing with these problems (Chen, 2019) [82] or to the difficulty of finding indicators to measure social phenomena, which hinders the application of other methodologies.

Contrary to traditional approaches, this research sought to transform these constructs into measurable variables. Through a theoretical review, dimensions were created that led to the statistical demonstration of the relationship between coexistence and otherness (as dependent and independent variables respectively) from a high significance mathematical model for use in applied research.

Regarding the creation and statistical validation of a scale to measure both variables, it is necessary to refer to some previous works, such as those of Gilat, Gindi & Sedawi-Massri (2020) [83], who addressed the issue of the sense of belonging in a Jewish community.

The study concluded that several teachers within this community did not perceive their Arab background as a problem, although some others stated that “it was as if other teachers ignored their ‘otherness’“ (Gilat, et al., 2020. p. 11) [83]. Expanding on this finding, they were able to identify a sense of belonging as a dimension of coexistence.

Although this assessment coincides with that of the present study, it should be noted that they used otherness as a concept and, moreover, as a referential framework to approach coexistence. Obviously, they did not have a measurement scale that could have been useful in this and other similar studies.

Another research on territoriality in Serbian and Bulgarian communities took identity and belonging as dimensions (Berceanu & Popa, 2022) [84]. An important statement of the findings is that ‘the First World War separated two countries or two states, but it cannot break the identity of the intimate connection between the inhabitants’ (p.10).

This statement, although powerful and well supported, is the result of a value judgement made by the authors, who also did not have a measurement scale. Nevertheless, it should be noted that this is a consistent vision in the way coexistence and otherness (and especially identity) are approached in this research.

## Conclusions

The first thing to note is that there is simply no scientific precedent for such a scale. This is important because the rightness or wrongness of the measurement of these variables can affect the validity of data and research results. Therefore, the correspondence between the measurement results and the reality of the phenomenon being studied could be lost (or overlooked). This is why the validation process is necessary.

The results show that traditionally four ways of interpreting the relationship with the Other have been defined, i.e., as stranger or foreigner, object, equal or otherness. However, the statistical analysis of the present work enabled the delimitation of only two dimensions for the variable otherness: (stranger or foreigner / equal).

The first dimension subsumed the questions constructed for the category of the other as an object, perhaps because people generally perceive the other as a stranger who may also appear harmful and potentially dangerous.

Because of this reasoning, the two concepts become associated. The others, the strangers, tend to be reduced to a number or a nickname, deprived of their subject quality, turned into an “immigrant”, a burden, or an obstacle.

The second dimension was that of “equal”, which refers to the other as a friend, as a kind person with whom a social relationship can be established. It is striking that the dimensions to which the work was reduced represent a polarity, i.e., the extremes of the other as stranger/alien vs. the other as equal. This is an important finding as it shows that the subjects’ perceptions oscillate between these two positions for social bonding.

In summary, the results showed that the constructed instrument measures otherness from the dimensions of the other as stranger/alien and the other as equal, which proved to be an instrument with a reasonable degree of reliability that responds to the dimensions to be assessed. This makes the use of the instrument valid.

## Data Availability

The datasets generated and/or analysed during the current study are not publicly available due to commitments to the institution that financed the research but are available from the corresponding author on reasonable request.
